# Udder, Claw, and Reproductive Health in Genomic Selection of the Czech Holstein

**DOI:** 10.3390/ani14060864

**Published:** 2024-03-11

**Authors:** Zuzana Krupová, Eva Kašná, Ludmila Zavadilová, Emil Krupa, Jiří Bauer, Marie Wolfová

**Affiliations:** 1 Institute of Animal Science, 10400 Prague, Czech Republic; kasna.eva@vuzv.cz (E.K.); zavadilova.ludmila@vuzv.cz (L.Z.); krupa.emil@vuzv.cz (E.K.); j-m-wolf@gmx.de (M.W.); 2 Czech Moravian Breeders Corporation, Genetic Evaluation, 25209 Hradištko, Czech Republic; bauer@plemdat.cz

**Keywords:** selection index, economic value, clinical mastitis, claw diseases, retained placenta, metritis, ovarian cysts

## Abstract

**Simple Summary:**

The enhanced selection index proposed in the study incorporates new health disorder traits related to udder, claw, and reproduction in addition to the current selection traits. Genetic parameters and breeding values for the traits were evaluated using linear animal models and single-step genomic best linear unbiased prediction (ssGBLUP) methods. The economic weights (EWs) for the new health disorders were calculated using the enhanced bioeconomic model implemented in the computer programme EWDC (i.e., economic weights for dairy cattle), which is versatile and can be applied for various breeds, farm management, and conditions. The use of the enhanced selection index mostly allowed favourable selection progress in terms of the new and current breeding objective traits and could be acceptable for local breeders.

**Abstract:**

The aim of this study was to construct an enhanced selection index using the genomic and economic parameters of new health disorders and current production and functional traits. Genomic evaluation for the incidence of clinical mastitis (CM), three claw disease traits, retained placenta (RET), metritis (MET), and cystic ovaries (CYS) was performed using linear animal models based on producer-recorded data. Good correlations among the health disorders were found, and their heritability estimates did not exceed 7%. Economic weights (EWs) for the health disorders were EUR −132.10 for CM, EUR −128.87 for overall claw diseases, EUR −52.10 for RET, EUR −80.48 for MET, and EUR −16.16 for CYS. These EWs indicate changes in the present value of the annual profit per cow when increasing the incidence of the traits by one case per cow year. Selection using the enhanced index resulted in favourable responses for most of the new health disorders (e.g., −0.001 and −0.006 cases of RET and MET per cow year, respectively), and also in the current breeding objective traits (+49 kg of milk, −0.02% of calf losses). An index contribution of 7% for the new health disorders was assessed as acceptable for the breeders.

## 1. Introduction

Protecting animal health and welfare in the context of sustainable food systems and inextricable links between healthy people, healthy societies, and a healthy planet are defined as some of the comprehensive challenges in the Farm to Fork Strategy [1]. One of the significant public health issues addressed in this strategy is antimicrobial resistance, which can be transmitted between humans and animals through the food chain [2]. Adequate prevention and only the necessary use of antibiotics in veterinary (and human) treatment are generally recommended for resistance inhibition. Therefore, efficient, healthy, and safe livestock production is crucial for food safety and public health.

Genetic selection and breeding programmes enhance improvements in animal health and output [3]. Genomic selection has been developing rapidly in recent decades, which is an ensemble of methods to estimate the breeding values of individual animals on the basis of genome-wide single-nucleotide polymorphism genotype information [4]. Selection of a robust dairy cow that is resistant to diseases, high producing, conceives easily, and produces healthy calves is highly desirable and results in profitability [5]. Moreover, the presence of diseases and associated reproductive disorders becomes more important in the context of global changes and thermal stress [6]. Therefore, current dairy cattle breeding is focused on production and functional traits, including indirect (longevity, survival) and direct (clinical mastitis, hoof disease, and reproductive and metabolic disorders) health traits (e.g., [5,7]). Some of the practical consequences of advanced animal health and resistance to diseases, e.g., mastitis, are improved milk quantity and quality, improved animal welfare, and optimal use of antimicrobials [8]. To address these issues, the sale of antibiotic veterinary medicinal products for food-producing animals nearly halved across Europe in the last decade and should be further reduced (to 59 mg/population correction unit settled by the 27 European Union Member States; [2]).

In this framework, the Czech Republic is among the countries with below average use of medicines per farm animal unit (50 mg in 2021; [2]). Further, a wide-ranging selection of the local Holstein population has been performed for three decades, covering the production, reproduction, indirect health, and exterior traits [9,10]. Genomic evaluation of Holstein cattle is routinely performed by the single-step GBLUP method. Most of these traits are also validated for Interbull genomic multi-trait across-country evaluation and their description is available on the website of Plemdat (in Czech [10]) and the Interbull website (in English [11]). Recently, the first calculations of the economic weights (EWs) and breeding values for udder [12] and claw health disorders [13] were outlined. Since then, the selection criteria of the local dairy population have been adjusted to unify selection in the sire and dam population [10]. Estimations of breeding values for the direct health traits (clinical mastitis and claw disorders) have been updated [14,15] and officially introduced into the national genomic evaluation. For reproductive disorders, the methodology for the estimation of genomic breeding values (GEBVs) has been established [16] but is not yet applied routinely. Moreover, all these direct health disorder traits have not yet been implemented in the overall selection index of the Holstein breed (denoted as SIH) [10]. All of these circumstances have created the possibility for enhanced selection for animal health, which is timely and relevant for the Czech Holstein breed.

The aim of the current study was to construct an enhanced SIH reflecting the genomic and economic parameters of new health disorder traits related to udder, claw, and reproduction and containing production and functional traits already considered in the Czech Holstein selection. For this purpose, population data were assessed and determined to (1) estimate genetic and genomic parameters for all the traits of interest in the Czech Holstein breed and (2) enhance the bioeconomic model and related programme EWDC (i.e., economic weights for dairy cattle) to newly calculate the economic weights for reproductive disorders.

## 2. Materials and Methods

The enhanced SIH index is based on the estimated genomic parameters and calculated economic weights (EWs) of the appropriate traits included in the breeding objective and those used as selection criteria in SIH.

### 2.1. Breeding Objective and Selection Criteria

Current breeding objective traits (10 in total) of the purebred Czech Holstein population are related to milk quantity and quality, reproduction, survival of calves and cows (productive longevity), growth, and udder health disorders (clinical mastitis, CM). In the SIH [10], 17 traits are currently involved as selection criteria, as mentioned above. In the present study, four health disorder traits (namely, incidences of overall claw disease (OCD), retained placenta (RET), metritis (MET), and cystic ovarian disease (CYS)) were added to the breeding objective. SIH index was enriched by GEBVs estimated for seven health disorder traits. In addition to the udder (CM) and reproductive health (RET, MET, CYS) mentioned above, the OCD trait selection criteria were specified there as infectious digital disorders (IDD) and non-infectious claw horn lesions (CHL). The lists of breeding objective traits and selection criteria are presented in Table 1 and Table 2, respectively. Details of the definition and evaluation of udder and claw health traits have been outlined in previous studies [12,13]. Therefore, the genomic multi-trait animal models implemented recently for CM and claw disorders [14,15] and the information on the estimation of GEBVs and EWs for the newly included reproductive disorders will be primarily presented in the following text.

### 2.2. Genomic Parameters

The genetic parameters and GEBVs were predicted in routine genomic single-step GBLUP evaluation provided by the Genetic Evaluation group (i.e., Plemdat) of the Czech Moravian Breeding Corporation (CMBC). CMBC is authorised by Czech government for independent official genetic evaluation of farm animals in Czech Republic. The exception of routine evaluation was reproductive health disorder traits (RET, MET, CYS) estimated by the Institute of Animal Science and will be soon implemented in routine. For both evaluations, the common datasets were used containing about 2,750,000 animals and 68,783 genotypes (5612 bulls registered for breeding and 63,171 cows and heifers). Performance of Holstein cattle has been recorded for routine genetic evaluation of production, reproduction and exterior traits for more than thirty years and health traits records for routine have started in 2017. Pedigrees of Holstein cattle have been fully recorded since first commercial use of imported Holstein semen in 1990 in accordance with Holstein Cattle Breeders Association of the Czech Republic (HCBA). The records and genotypes are property of individual farmers, HCBA and CMBC which is authorised by government also for data administration.

Subsequently, brief descriptions of the datasets and the methodology used for health disorder breeding value predictions are presented in Table 3 and in the following text, respectively. Health traits were defined and recorded by producers/farmers in accordance with the International Committee for Animal Recording (ICAR) Guidelines [17] following the Czech ICAR Central Health Key [18]. All health disorder traits were expressed as binary, i.e., as their presence (1) or absence (0) recorded in a given lactation period, i.e., from calving to 14 days of lactation for RET to 150 days of lactation for CM and other reproductive disorders, and to 305 days for udder and claw diseases [14,15,16]. The somatic cell score (SCS) was calculated as the average value per lactation. A detailed definition and description of the health disorder traits were presented in previous studies [14,15,16].

The Holstein Cattle Breeders Association of the Czech Republic provided all health, production, reproduction, and linear type traits, and genomic data, including pedigree. The CM, IDD, CHL, and OCD datasets included 121,200, 60,109, 9983, and 69,847 Holstein cow lactations, respectively. Cows calved between 2017 and 2023 in 130 herds (CM), 97 (IDD), 36 (CHL), and 106 (OCD) herds, respectively. Data for reproductive disorders were recorded between July 2017 and June 2023 in 120 (RET), 145 (MET), and 103 (CYS) herds including records from 144,921 (RET), 215,187 (MET), and 142,251 lactations (CYS). The average lactation incidences of udder, claw, and reproductive disorders are presented in Table 1.

Genomic data for 68,783 Holstein bulls and cows genotyped with the Illumina Bovine SNP50 BeadChip (50K, Illumina Inc., San Diego, CA, USA) were also used. Genotypes were coded as 0, 1, 2, and 5 (missing SNP) for calculation of the genomic relationship matrix (G). Monomorphic SNP, animals with genotype call rates <0.90, SNP with call rates <0.90, SNP out of Hardy–Weinberg equilibrium, with minor allele frequency <0.05, and individuals with parent–progeny conflicts were excluded from the evaluation. After quality control, the number of genotyped animals was 68,783, and the number of effective SNP was 35,517. QCF90 software ver. 1.2.0 was used for SNP quality control [19].

#### Animal Model

For prediction GEBVs of CM, multi-trait animal linear models were used that also analysed the SCS per lactation and udder exterior traits (udder depth, udder width, suspensory ligament, and udder subjective score in %). For prediction of the claw disease trait GEBV, multi-trait animal linear models were also used, that analysed the foot angle and locomotion score with IDD, the feet and legs score, rear legs from side view, foot angle and locomotion score with CHL, and the feet and legs score and locomotion score with OCD. The reproductive disorders were analysed with single-trait animal linear models.

In simplified notation, the models can be written as follows:

Health disorder traits and SCS = HYS + PA + pe + a +e(1)

Exterior traits = HYS + C + age + age^2^ + dim + dim^2^ + a + e (2)

where the effects were as follows: HYS—a fixed effect of herd*year*season at calving/scoring; PA—a fixed effect of cow parity for reproductive disorders and cow parity*age at calving (primiparous) or cow parity*calving interval (multiparous) for the other disorder traits; C—a fixed effect of a classifier; age and age^2^—fixed linear and quadratic regression of age at calving; DIM and DIM^2^—fixed linear and quadratic regression of days in milk at scoring; a—random animal additive genetic effect; pe—random permanent environmental effect; and e—residual effect.

The models for health disorder trait evaluations in matrix notation can be written as follows:

**y** = **Xβ** + **Z_pe_pe** + **Z_a_a** + **e**, (3)

where **y** is a vector of observations (presence or absence of the disease/disorder within parities within cows); **β** is a vector of systematic effects; **pe** is a vector of random permanent environmental effects of a cow; **a** is a vector of random animal additive genetic effects; **e** is a vector of random residuals; and **X**, **Z_pe,_** and **Z_a_** are the corresponding incidence matrices.

Model assumptions were as follows:

*a ~N*(0,***H****var*(*a*)), where *var*(*a*) is the direct additive genetic variance and **H** is the pedigree–genotype relationship matrix [20,21]; *pe ~ N*(0,***I****var*(*pe*)), where *var*(*pe*) is the variance due to the permanent environment, **I** is the identity matrix, and *e ~N*(0;1).

The models for exterior traits were:

(4) $$y=X\beta +Z_{a}a+e$$

Variance components were estimated using the residual maximum likelihood method with an average information algorithm, as implemented in AIREMLF90 [19]. Heritabilities of all health traits were calculated as follows:

(5) $$h^{2}=\frac{var(a)}{var\left(a\right)+var\left(pe\right)+var(e)}$$

and for exterior models:

(6) $$h^{2}=\frac{var(a)}{var\left(a\right)+var(e)}$$

The heritabilities were validated in the Interbull genomic evaluation, which is available at [11] for Czech Republic.

GEBVs were estimated with the single-step GBLUP method, as implemented in BLUP90IOD2 [19]. The reliabilities of GEBVs were approximated with ACCF90GS [22]. GEBVs were reversed in sign so that higher values reflected better resistance to the disease/disorder, i.e., lower incidence of health disorder traits. They were expressed as relative breeding values (RBVs) with mean = 100 and standard deviation = 12, for base bulls born in 2010. The associations between all traits were quantified using Pearson correlations between RBVs of animals born since 2000.

### 2.3. Economic Weights of Breeding Objective Traits

Economic weights (EWs) of traits expressing economic importance were calculated for the breeding objective traits listed in Table 1 with respect to the purebred production system of Czech Holstein cattle with integrated fattening of bulls. The programme EWDC (i.e., economic weights for dairy cattle), which is a part of the programme package ECOWEIGHT [23], was used for this calculation. The new reproductive disorder traits, i.e., the average incidences of RET, MET, and CYS, were included in the bioeconomic model of EWDC in 2023. These incidences are defined as the number of cases of the relevant disease per cow year averaged over all lactations. For the present calculation, the production parameters of the breed were taken from the Annual Report [24] and from the database provided by the HCBA. Mean values of the breeding objective traits for the year 2022 are presented in Table 1. Basic economic parameters of Holstein cattle farms were taken from the study of Syrůček et al. [25].

The general methods applied in EWDC for the calculation of trait EWs were described in detail previously [12,23]; therefore, only the specific approaches applied for the new reproductive disorder traits will be detailed here.

As for all other traits evaluated by EWDC, the EWs of the average incidence of RET, MET, and CYS express the changes in the present value of the annual profit per cow when the incidence of these traits increases by one case per cow year. The changes in profit are caused by losses in revenue due to discarded milk from cows treated with antibiotics (only for RET and MET) and by increased costs for drugs and for veterinarian and herdsman time connected with treatment of all three named diseases.

Base input parameters for the calculation of EWs of all five health disorders, i.e., reproductive, udder, and claw diseases (valid for the year 2022) are presented in Table 4. These inputs represent the costs for veterinary procedures and treatments applied at Czech Holstein cattle farms and were obtained through personal communication with herd veterinarians and farmers. The medicines commonly used on farms for treatment of the evaluated diseases were authorised and published by the Institute for State Control of Veterinary Biologicals and Medicines [26]. The costs for drugs were based on the official prices of veterinary medical products [27].

**Figure 1:**
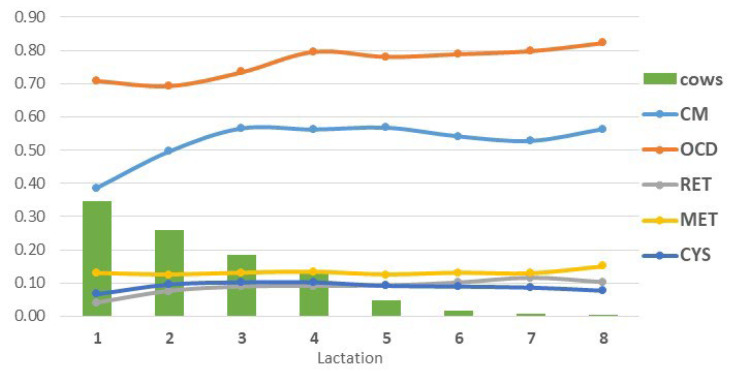
Incidence rates of udder, claw, and reproductive diseases and ratio of cows in lactations 1 to 8. CM—clinical mastitis, OCD—overall claw disease, RET—retained placenta, MET—metritis, CYS—cystic ovarian disease. Based on authors’ calculations from the database provided by the Holstein Cattle Breeders Association of the Czech Republic.

**Figure 2:**
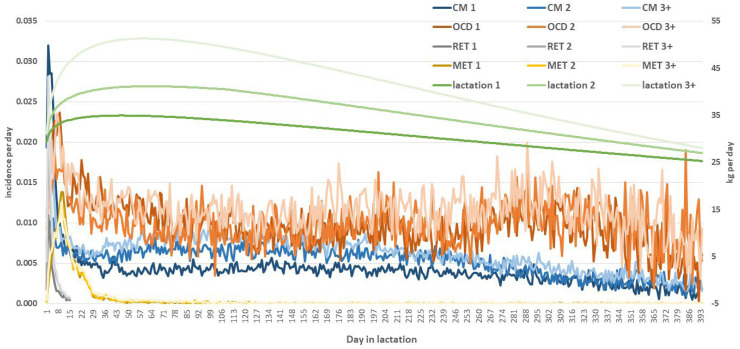
Daily milk yield (in kg) and daily incidence^1^ of udder, claw, and reproductive diseases treated with antibiotics in different lactations. ^1^ Number of cows having the disease treated with antibiotics; their milk had to be discarded on day *i* of lactation *l* divided by the number of cows in the herd on day *i* of lactation *l*. CM—clinical mastitis, OCD—overall claw disease, RET—retained placenta, and MET—metritis; 1—first, 2—second, and 3+—third and subsequent lactations. Based on authors’ calculations from the database provided by the Holstein Cattle Breeders Association of the Czech Republic.

Disease incidences in Holstein cows in lactations 1 to 8 are presented in Figure 1. These include diseases treated with and without antibiotics. The only exception was CYS incidence, where only non-antibiotic (i.e., hormonal) treatment was applied. In the case of the antibiotic treatment of CM, OCD, RET, and MET with a withdrawal period for the delivery of milk to dairies, the amount of discarded milk was estimated from the daily incidence of diseases treated with antibiotics with a withdrawal period in the particular lactation and from the lactation curve for this lactation. The lactation curves and the daily incidences of diseases treated with antibiotics in corresponding lactations are presented in Figure 2. The losses in revenue from milk were then calculated, assuming an average milk price of EUR 0.426 per kg [28]. This milk price represents the mean of the last three-year period because relatively high price volatility (varying from EUR 0.357 to 0.537) occurred in recent months [28].

A detailed description of the procedure for the estimation of EWs for mastitis incidence can be found in the study by Wolfová et al. [12], and for claw disease incidence a description can be found in the study by Krupová et al. [13].

### 2.4. Selection Index and Response

By constructing an enhanced SIH index, the expected genetic and economic selection response in the breeding objective traits and reliability of the SIH were computed. To measure the individual effects of the new group of health disorder traits (udder, claw, and reproductive) of the enhanced selection, three variants of the SIH were investigated:

(1) Current SIH + udder (CM);
(2) Current SIH + udder + claw (CM + OCD + IDD + CHL);
(3) Current SIH + udder + claw + reproductive (CM + OCD + IDD + CHL + RET + MET + CYS).

In these variants, two alternatives for the weighting of traits were applied:

- Optimal weighting of all traits to maximise the selection response in the breeding objective traits and the reliability of selection (labelled as “opt”);
- Weighting the current traits equal to the current SIH index by default and taking space for the new health trait(s) in indices from the optimal relative contribution/proportion (labelled as “def”);
- In variant 3, the breeders’ preferences (represented by the HCBA) for the weighting of selection traits were applied. Given the partial calculations the following contribution was settled by them as acceptable: current traits equal to the current SIH index, by default up to 93%, and taking 7% for the new health disorder traits from the optimal relative contribution/proportion (labelled as “pref”).

In total, seven index variants and alternatives of SIH index were evaluated in the study (1 opt, 1 def, 2 opt, 2 def, 3 opt, 3 def and 3 pref).

By providing calculations in all index variants and alternatives, the general principles of the selection index theory were applied. The genetic selection responses in the breeding objective traits were calculated assuming a standardised selection intensity of 1.0 and 4 selection paths (sires of sires, sires of dams, dams of sires, and dams of dams). Calculations of the selection response have been presented in detail by Přibyl et al. [9] and Krupová et al. [13]. The economic response represents the sum of the economic response in all breeding objective traits, taking into account the appropriate trait genetic response and the trait EW. For monetary units, the average annual exchange rate of EUR 1 = EUR cents 100 = CZK 24.565 was applied (https://www.kurzy.cz/kurzy-men/historie/EUR-euro/2022/ (accessed on 24 September 2023)).

## 3. Results

### 3.1. Genomic Parameters

Genetic standard deviations of the breeding objective traits are given in Table 1; correlations among the GEBVs of the selection criteria, reliabilities of the GEBV estimation, and trait heritability are presented in Table 2. The heritabilities of production traits were moderate with max. 0.380 for fat yield and content. The longevity had a heritability of 0.223. The lowest heritability was estimated for a conception rate of 0.044. The heritabilities of exterior traits ranged from 0.07 for locomotion to 0.324 for teat length. The heritability estimates for all health traits were low and did not exceed 7%. The distribution of GEBV estimates was close to normal with a mean = 0 and genetic standard deviation from 0.01 (RET, CYS) to 0.04 (OCD) (Table 1). The lowest mean reliability of GEBV estimates was found in RET (0.099); the highest was found in CM (0.239; Table 2). A strong positive correlation was calculated between OCD and IDD (0.853); a moderate correlation was obtained between RET and MET incidence (0.410; Table 2). Other correlations among health traits were weak and mostly positive, with the strongest association between MET and IDD incidence (0.163).

Concerning the correlations between health disorder traits and other traits in SIH (Table 2), a moderate correlation was found between CM and longevity (LONG; 0.388), CM and somatic cell score (SCS; 0.298), and MET and LONG (0.264). IDD showed higher correlations with locomotion (LOC; 0.205) and legs score (LEGS; 0.201); CHL was related especially to foot angle (FA; −0.322). Each of the three assessed foot and claw disorders (OCD, IDD, CHL) showed different correlations to the other analysed traits. Therefore, it could be said that they differ from each other, and their separate inclusion in the index is justified. The relationships between MET and conception rate (CR; 0.186), CM and udder depth (UD; 0.160), and CM and fore udder attachment (FUA; 0.127) should also be mentioned.

### 3.2. Economic Weights

The economic weights (EWs) calculated for breeding objective traits of the Czech Holstein population are presented in Table 5. The absolute EWs per cow and year ranged from EUR 0.023/day to EUR 520.1/% for the service period and milk protein content, respectively. Concerning the health disorder traits, the lowest EW was estimated for the incidence of CYS (EUR −16.16/case) and the highest for CM incidence (EUR −132.1/case). These values mean that progeny of the selected bulls with better breeding values for CYS and CM incidences would increase the farm profit within the investment time period of 25 years by EUR 16 and 132 per cow and per year for each decrease in the CYS and CM incidence by one case/cow year, respectively.

From the total economic losses associated with the particular health disorders, the highest proportion usually receives a share of reduced revenues due to discarded milk (e.g., 40% and 73% for CM and OCD, respectively). This source of losses did not occur in the case of CYS disease, because no antibiotic treatments and thus no withdrawal period of milk were applied. The next most important sources of losses were additional costs for drugs, ranging from 21% (CM) to 42% (RET). Other costs related to veterinary and herdsman labour (in the case of CM, these also relate to depreciation costs for separate milking machines) represented from 6% (OCD) to 39% (CM) of the total economic losses caused by health disorders. This source of loss was the highest in the case of CYS (64%) caused by the above-mentioned specifics in treatment.

### 3.3. Selection Index and Response

**Figure 3:**
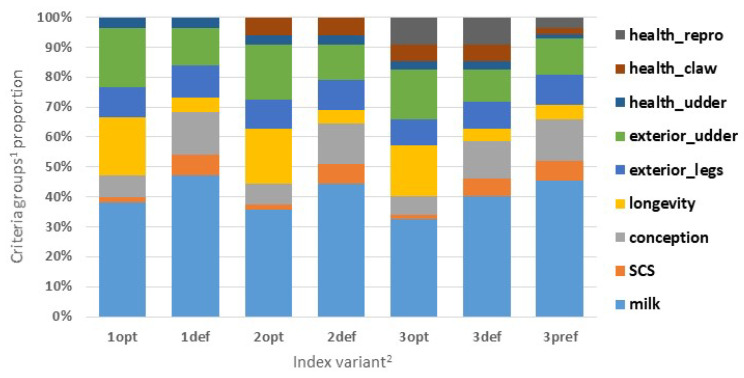
Proportion of the selection criteria groups^1^ (%) in the index variant^2^. ^1^ Selection criteria groups: milk (%F, kgF, %P, kgP), SCS (somatic cell score), conception (CR), longevity (LONG), exterior_legs (RLRV, FA, LOC, LEGS), exterior_udder (FUA, FTP, TL, UD, RUH, CLI), health_udder (CM), health_claw (OCD, IDD, CHL), health_repro (RET, MET, CYS). For descriptions of the criteria abbreviations, see Table 2 and the Section 2. ^2^ Details of the investigated index variants are given in Table 5 and in the Section 2.

The proportion of the selection criteria in the enhanced SIH variants is presented in Figure 3. The traits were divided into nine main groups to present the impact of the new direct health traits on the index construction with respect to the current sub-indices of SIH. The average contributions of udder, claw, and reproductive traits in the enhanced SIH were 3%, 5%, and 7%, respectively. The highest proportion remained for milk traits (41%), followed by the exterior of the udder (15%); the conception, longevity, and exterior of the feet and legs (10% each); and SCS (4%). For the optimal index variants, a higher contribution of longevity (e.g., 17% vs. 5%) and lower proportion of SCS (2% vs. 7%) and conception rate (6% vs. 14%) was determined compared to the default weighting of traits (index 3 opt vs. 3 pref).

The selection gains in all of the Czech Holstein breeding objective traits and the overall economic response expected under the current and enhanced variants (and alternatives) of the SIH index are presented in Table 5. The genetic selection responses in the new udder, claw, and reproductive traits were positive. This means that a slight decrease in disorder incidences ranging from −0.001 (RET) to −0.006 (MET) cases (both in optimal variant 3 considering all new health traits in selection) was calculated. The only exception was CYS, where a slight increase in incidence (+0.001 cases, in all alternatives of variant 3) would be expected in the enhanced selection. Genetic responses in the rest of the breeding objective traits were mostly favourable (e.g., +49 kg of milk, +0.03% of milk fat, −0.02% of calf losses, and +0.07 year, i.e., +27 days of productive longevity of cows, all in default alternative of variant 3).

Comparing the two approaches for the weighting of traits in the index variants, the overall economic response (in EUR) and reliability of the SIH (in %) were higher when the optimised rather than the default proportion of the selection criteria was applied (e.g., EUR 40 vs. EUR 28 and 44% vs. 36% for SIH index variant 2 and alternatives “opt” and “def”, respectively; Table 5).

## 4. Discussion

### 4.1. Genomic Parameters

Low heritability estimates for health traits were consistent with results from other studies that applied a linear animal model to producer-recorded health data (e.g., [5,29]). Generally, the heritability of health traits is low and typically does not exceed 10%. Nevertheless, genetic selection for improved health is possible, as experience in the Nordic countries has shown (e.g., [30]).

The correlations among trait GEBVs estimated in the present study are consistent with our previous results [16] calculated using slightly different data (longer lactation periods used for MET and CYS recording; a different sample of bulls with RBV). The mostly positive correlations among the health disorder traits allow us to assume that selection for improving resistance to one of the diseases would lead to favourable selection responses in others. This, according to Heringstad et al. [31], indicates the existence of a general disease resistance factor with a genetic component, such as the major histocompatibility complex.

Although cows with higher genetic merit for production are expected to be more susceptible to diseases, the correlations between the GEBVs of the evaluated disease incidences and of milk production traits (except protein yield) have been positive, i.e., favourable, in some studies (e.g., [5,32]). The authors Koeck et al. [32] also reported moderate negative correlations between milk/protein yield and CM, CYS, and lameness. Better resistance to reproductive disorders was correlated with higher conception rates and better longevity [5,32]. As expected by Jamrozik et al. [33], CM was related to SCS (with a correlation of 0.298) and also to LONG (0.388). The moderate correlation between CM and LONG suggests that CM is one of the main reasons for the culling of cows. All foot and claw disorders analysed in the present study were related to LEGS, FA, and LOC, and LOC is one of the most essential conformation traits associated with foot and claw health [34]. Moderate correlations between FA, feet and legs score, and several foot and claw disorders were also found by Koenig et al. [35]. OCD and IDD were correlated with CR and LONG. This was in accordance with the findings of Buch et al. [36], who published genetic correlations between foot and claw disorders and several reproduction traits, while Charfeddine and Pérez-Cabal [37] found a significant effect of claw health on LONG in dairy cows.

### 4.2. Economic Weights

When discussing the economics of dairy health disorder traits calculated in the present study and published in the literature, some specifics such as the economic effect expression and the calculation methodology should be taken into account.

#### 4.2.1. Expression of the Economic Effect

The economic importance (effect) of dairy cattle health disorders in published studies [38,39,40] and in our study usually quantifies the supplementary costs needed for medicines and time (labour) spent for the herdsman (farmer) and veterinary treatment, along with reduced revenues arising during the treatment of animals with antibiotics requiring withdrawal periods (i.e., due to discarded milk). In addition to the direct economic consequences, other losses related to the evaluated health trait have often been considered. The effect of disease occurrence on milk yield in the rest of the lactation and on the subsequent parities (even with an associated reduction in feed intake), on the reproductive ability of cows (conception rate, days open), on the probability of the presence of other health problems, and on the culling risk of cows are considered in published studies. In the context of the trait EWs calculated for selection purposes, such correlated effects should not be considered, to avoid double counting. In the case of structural herd interactions, some correction for effects from alterations in correlated traits using multiple regression analysis could be applied when calculating the trait EWs [41].

Therefore, the calculation of EWs for the health disorder traits of the Czech Holstein population presented in Table 5 was based on the quantification of direct effects on the net profit per cow and year, i.e., only the changes in costs and revenues directly associated with the treatment of health disorders in dairy herds were taken into account. The correlated effects on the breeding objective traits evaluated simultaneously (and vice versa) were not considered. For example, the impact of RET incidence on milk yield during the rest of the lactation (and on further lactations) or the effect of milk yield on the reproductive success (conception rate) of cows was not taken into account in the trait EW.

#### 4.2.2. Calculation Methodology

Calculation of the trait EW presented in the current study was achieved via the bioeconomic model of EWDC [23] similar to studies mentioned above [38,39,40]. According to Hirooka [42], bioeconomic models facilitate expressing the proper and comprehensive impact of the trait change on the economic performance of the system. This tool is therefore valuable for the appropriate economic weighting of traits and for the preservation of the enhanced selection response in the genomic area. Also, Hogeveen et al. [8] stated that the methodological progress provided recently in bioeconomic models enables the use of biological aspects when evaluating the economics of production diseases.

Recently, Robics et al. [38] estimated the losses associated with foot disorders in dairy herds. Average annual losses associated with a lame cow and a cow affected by digital dermatitis were almost three times higher compared to our calculation for OCDs, mainly due to considering the associated milk losses before and after the occurrence of lameness and the increased risk of reproductive disorders and culling of cows. Moreover, the economic outcomes expressed by these authors as a gross margin (i.e., without operating expenses) could play a role. Similarly, Liang et al. [39] estimated the losses associated with seven clinical diseases (as well as others for CM, lameness, MET, and RET) in US dairy farms that were two to four times higher than in our present calculation. Subtracting the secondary losses (caused by correlated effects), the direct economic consequences of the health disorders would be halved in the US study (to about USD 149, USD 137, and USD 97 for CM, MET, and RET, respectively). These values are much closer to the EWs calculated in our study for the same reproductive disorders (given in Table 5). The remaining difference could be caused by the considerably higher price for veterinary services in the US compared to the Czech Republic.

Economic values of the udder, claw, and reproductive health disorders calculated for Nordic Holstein populations by Kargo et al. [40] were about three times higher compared to our results. Economic values were higher in spite of the fact that only direct economic costs (for medicines and veterinarian and farmer time) without losses for discarded milk were taken into account. Higher veterinarian costs including fees and medicines and farmer time per treatment could be the reasons for such differences. Similarly, the direct economic effects of CM, lameness, and MET calculated for the German Holstein population [41] indicated about two times higher economic values, mainly caused by higher costs for treatment and losses from milk withdrawal.

#### 4.2.3. Discount Rate

To calculate the economic importance of traits and their expression in the progeny of the selected bull for the future investment period (of 25 years), the discount rate has been considered [12,43]. The main aim is to take into account the delay in the expression of traits (and associated revenues and costs) over the animal’s life. The discount rate is generally a combination of the average yearly interest and inflation. In 2022, the historically high inflation in the Czech Republic (15.1%; [44]) and in Europe (9.2%; [45]) was about triple that of the previous years. Similarly, an increase was recorded in the interest rate of loans [46]. Based on the Statista [47], the EU inflation rate declined to 3.4% in December 2023. Therefore, in the current study, all revenues and costs associated with the evaluated traits were discounted with an annual rate of 2%, which is comparable to previous studies (ranging from 2% to 7%; [12,13,43]. Similarly, a rate of 2.11% was used in the stochastic simulation study by Liang et al. [39] when calculating the losses related to clinical disease incidences in dairy herds.

#### 4.2.4. Versatility of the Model

The above-mentioned simulation models [38,39,40], as well as the model presented in our current and previous studies (cited in this paper), belong to the normative approaches. They offer some flexibility to users and can be applied to various production parameters, populations, and biological features when evaluating farm economics and the economic impact of production and functional traits on farm efficiency. The input files (parameters) of EWDC [23] are settled generally to facilitate the variation in disease incidence (within and over lactations), disease severity, veterinary (treatment) procedures (with and without antibiotics), herd status, and management. The model enables us to settle the daily incidence of cows treated with antibiotics with a withdrawal period within individual lactations (days in milk) if needed. Eventually, zero values could be inputted by the user when such treatment is not applied in the herd. Likewise, as in the study of Liang et al. [39], no antibiotic treatment was considered when calculating the losses associated with RET incidence. Similar to Viking Genetics [48] and Kargo et al. [40] the economic importance of specific diseases/disorders or their groups can be alternatively calculated using the appropriate data in the existing input files of the EWDC programme. As with the study of Robics et al. [38], various strategies and severity of individual diseases/disorders and their treatment could be simulated in the EWDC model to express the variability of their impact on the farm economy, and to present farmers the economic consequences. These options show the model and programme to be versatile and applicable for various dairy cattle breeds, farm management, and conditions.

### 4.3. Enhanced Selection

A favourable selection response with respect to most of the udder, claw, and reproductive disorder traits calculated for enhanced selection of the Czech Holstein breed (Table 5) assumes the improvement of resilient animals in further generations with measurable economic benefits (reducing the use of medicines; animal treatments; and milk losses). The slightly unfavourable selection gain calculated for CYS disorders (+0.001 cases) may be based on the mostly negative correlation of this trait with milk and exterior selection traits (e.g., −0.114 with kg of milk fat and −0.121 with udder depth; Table 2). The groups of milk and exterior traits represent an important part of the enhanced SIH variants (41% and 24%, respectively; Figure 3); therefore, their improvement could lead to a slight increase in CYS incidence. Nevertheless, this relationship will be updated in the future with increased numbers of herds and lactations considered in the genomic evaluation to confirm the relationship.

Favourable genetic and economic selection responses expected for most of the current breeding objective traits of the Holstein breed (Table 5) are essential for the acceptability of the enhanced SIH by the local breeders and generally for the sustainability of the breed. The only exception is the unfavourable increase in mature weight (that varied from +0.67 to +1.11 kg in 2 opt and 3 def variants of the index, respectively). The main reason for this could be the generally known positive correlation of this trait with milk yield. Based on the local breeders’ preferences, 7% will be an acceptable contribution of the new health traits to the SIH. This is in agreement with the selection formulas applied in the Holstein breed across the world, where the contribution of health traits ranges from nearly 2% in the American TPI index [49] to 18% in the English £PLI index [50], to 20% in the Nordic NTM [48]. Likewise, in the Jersey breed, the genomic evaluation of various disorder traits of cows was developed to be implemented in animal selection and contribute to genetic progress [7].

The overall reliability of the enhanced SIH (40% on average; Table 5) was mainly a function of the reliability of the estimated GEBVs (ranging from 0.099 for RET to 0.560 for %F) and of the proportion of particular traits in the index (varying from 0.001 for RET to 0.270 for kgP). The overall reliability presented in the current study was somewhat lower than our previous estimates (60%) for the Czech Holstein breed [13]. This could be explained firstly by the different datasets of animals (bulls vs. whole population), later also considering animals with lower reliabilities of estimated GEBVs, and secondly, by the generally lower reliabilities of the newly included health disorder traits in comparison to the current selection criteria (e.g., as mentioned above for RET and kgP). Similarly, as suggested in the case of the correlations among traits, regular updating of the genomic parameters will be undertaken with an increased dataset in the future to update the population selection scheme. Nevertheless, in spite of the lower reliabilities of the GEBVs for the newly incorporated health disorders, the selection responses for these traits, as well as responses in the current breeding objective traits, were mostly favourable, promising desirable gains under the enhanced selection.

## 5. Conclusions

Genomic evaluation of direct health disorder traits currently showed low heritability of those traits and low reliabilities of predicted breeding values. Generally, favourable correlations among the health characteristics could be utilised by selection. The expected economic response and reliability of the investigated SIH alternatives were higher under the optimised selection than under the default and preferred weighting of index traits. Selection based on an index extended with traits characterising cow health resulted in favourable responses in most of the new health disorders, as well as in the current breeding objective traits. Considering the local breeders’ preferences, the acceptable contribution of the new health disorder traits would enhance selection and improve animal resilience in further generations, with measurable economic benefits.

## Figures and Tables

**Table 1:** Breeding objective trait characteristics in the Czech Holstein population ^1^.

Trait			Unit	Abbreviation	Mean	Genetic Standard Deviation
1	current	Milk yield per 305 d of lactation	kg	MY	10,544	562.6
2		Milk fat content	%	%F	3.87	0.187
3		Milk protein content	%	%P	3.38	0.101
4		Conception rate of cows	%	CR	85.5	2.49
5		Service period	day	SP	109	4.28
6		Losses of calves in rearing	%	CL	2.08	0.69
7		Age at first calving	day	AFC	375	9.61
8		Mature weight of cows	kg	MW	635	18.07
9		Productive lifetime of cows	year	PL	2.90	0.227
10		Clinical mastitis	cases/year of risk	CM	0.484	0.035
11	new	Overall claw disease		OCD	0.727	0.040
12		Retained placenta		RET	0.070	0.014
13		Metritis		MET	0.129	0.022
14		Cystic ovarian disease		CYS	0.088	0.012

^1^ Based on the authors’ calculation from the database provided by the Holstein Cattle Breeders Association of the Czech Republic.

**Table 2:** Selection index trait parameters ^1^ in the Czech Holstein population.

Trait ^1^	%F	kgF	%P	kgP	SCS	CR	LONG	RLRV	FA	LOC	LEGS	FUA	FTP	TL	UD	RUH	CLI	CM	OCD	IDD	CHL	RET	MET	CYS
%F	0.560																							
kgF	0.140	0.560																						
%P	0.739	0.010	0.533																					
kgP	−0.038	0.549	−0.008	0.533																				
SCS	0.031	0.004	−0.031	−0.010	0.487																			
CR	0.086	0.030	0.042	−0.024	0.074	0.343																		
LONG	0.078	0.047	0.058	0.004	0.485	0.323	0.544																	
RLRV	0.024	0.175	0.002	0.173	0.048	0.019	0.023	0.474																
FA	−0.004	0.059	−0.013	0.049	0.047	−0.033	0.086	0.324	0.347															
LOC	0.093	0.153	0.039	0.095	0.035	0.101	0.154	0.634	0.271	0.309														
LEGS	0.065	0.192	−0.009	0.135	0.036	0.109	0.245	0.703	0.551	0.784	0.362													
FUA	0.113	0.156	0.026	0.107	0.098	0.167	0.336	0.292	0.169	0.463	0.538	0.433												
FTP	0.010	0.135	−0.079	0.086	−0.033	0.073	0.042	0.104	0.093	0.400	0.428	0.466	0.442											
TL	−0.083	−0.020	−0.058	0.012	0.026	−0.045	−0.007	−0.014	0.050	−0.036	−0.019	−0.055	−0.127	0.478										
UD	0.069	0.131	0.005	0.089	0.137	0.216	0.456	0.249	0.189	0.452	0.556	0.802	0.488	−0.080	0.474									
RUH	−0.026	0.191	−0.130	0.149	0.036	0.171	0.292	0.299	0.141	0.449	0.584	0.662	0.528	−0.032	0.719	0.430								
CLI	−0.094	0.009	−0.127	0.012	0.018	0.012	−0.005	0.005	0.121	0.185	0.249	0.167	0.390	0.181	0.270	0.343	0.401							
CM	0.103	−0.048	0.130	−0.075	0.298	0.107	0.388	0.085	0.026	0.019	−0.011	0.127	−0.110	0.005	0.160	−0.075	−0.028	0.239						
OCD	0.051	0.039	0.032	0.001	0.019	0.116	0.129	0.071	−0.039	0.086	0.080	−0.016	−0.038	−0.006	−0.011	−0.031	−0.005	0.157	0.140					
IDD	0.066	0.066	0.043	0.026	0.005	0.132	0.161	0.046	−0.023	0.205	0.209	0.067	0.009	−0.026	0.061	0.045	−0.013	0.179	0.853	0.125				
CHL	0.033	0.015	0.028	−0.003	−0.020	0.093	0.036	0.103	−0.322	0.066	−0.042	0.103	0.077	−0.044	0.081	0.046	−0.076	0.087	−0.032	0.057	0.111			
RET	0.088	0.043	0.035	−0.009	−0.003	0.093	0.044	0.012	0.041	0.053	0.070	0.051	0.052	−0.056	0.052	0.030	0.014	0.003	0.055	0.066	−0.049	0.099		
MET	0.095	0.120	0.060	0.073	0.018	0.186	0.264	0.049	0.007	0.135	0.169	0.191	0.131	−0.038	0.215	0.196	0.002	0.114	0.076	0.163	0.088	0.410	0.144	
CYS	0.003	−0.091	−0.001	−0.114	0.002	0.021	0.006	0.110	−0.022	−0.059	−0.099	−0.172	−0.099	−0.003	−0.121	−0.130	0.004	0.090	0.160	0.131	0.007	0.032	0.002	0.104
h^2^	0.380	0.380	0.370	0.370	0.301	0.044	0.223	0.161	0.102	0.07	0.121	0.238	0.271	0.324	0.316	0.232	0.183	0.055	0.040	0.070	0.080	0.020	0.020	0.020

^1^ Reliability of genomic breeding value estimation (on diagonal), genetic correlations (under diagonal), and heritability (h^2^) of estimated breeding values of selection criteria: %F, kgF, %P, kgP—percentage and kg of fat (F) and of protein (P); SCS—somatic cell score; CR—conception rate; LONG—longevity; RLRV—rear leg rear view; FA—foot angle; LOC—locomotion; LEGS—legs; FUA—fore udder attachment; FTP—front teat placement; TL—teat length; UD—udder depth; RUH—rear udder height; CLI—central ligament; CM—clinical mastitis; OCD, IDD, CHL—claw diseases overall, infectious digital disorders, non-infectious claw horn lesions, respectively; RET—retained placenta; MET—metritis; CYS—cystic ovarian disease. Based on own calculation from the database provided by the Holstein Cattle Breeders Association of the Czech Republic.

**Table 3:** Descriptions of the datasets and characteristics of health disorder traits.

Trait	Abbreviation	Definition ^1^	Number of Animals ^2^
Clinical mastitis	CM	Visually abnormal milk secretion accompanied by signs of inflammation of udder tissue	73,664
Overall claw disease	OCD	All claw diseases comprising infectious digital disorders and claw horn lesions	39,553
Infectious digital disorders	IDD	Including digital and interdigital dermatitis; interdigital phlegmon and heel horn erosion	7829
Claw horn lesions	CHL	Including non-infectious disorders: ulcers, white line disease, horn fissures, and double sole	45,361
Retained placenta	RET	Non-repulsion of foetal membranes within 24 h after calving	85,510
Metritis	MET	Inflammation of the uterus	121,072
Cystic ovarian disease	CYS	Presence of a rounded structure with a diameter greater than 25 mm on one or both ovaries	82,267

^1^ Definition according to the ICAR Guidelines [17] and the Czech ICAR Central Health Key [18]. ^2^ Based on the authors’ calculation from the database provided by the Holstein Cattle Breeders Association of the Czech Republic. All health traits are expressed as cases/year of risk.

**Table 4:** Base parameters for the calculation of EWs for udder, claw, and reproductive disorder incidences ^1^.

Parameter	Unit	CM	OCD	RET	MET	CYS
Cost of drugs per case treated with antibiotics	EUR	28.21 ^2^	24.47	28.62	16.28	-
Cost of drugs per case not treated with antibiotics	EUR		16.57	3.01	18.73	3.95
Veterinarian’s time spent per average case	Hour	0.20	0.33	0.25	0.25	0.25
Herdsman’s time dealing with average case treatment	Hour	0.30	0.42	0.33	0.30	0.25
Depreciation cost for a separate milking machine	EUR/year and sick cow	1.55	-	-	-	-
Minimum/maximum proportions of diseases treated with antibiotics in a lactation		0.95 ^3^	0.24/ 0.36	0.42/ 0.49	0.10/ 0.13	-
Withdrawal period (min/max) for the delivery of milk to the dairy ^4^	Days	0/6.5	0/7	0/4	0/1	0
Average charge for veterinary service	EUR/hour	17.71				
Value of herdsman’s time	EUR/hour	10.58				

CM—clinical mastitis, OCD—overall claw disease, RET—retained placenta, MET—metritis, CYS—cystic ovarian disease. ^1^ Medical procedures and treatments applied at Czech Holstein cattle farms (personal communication with veterinarians). For monetary units, the average annual rate of EUR 1 = EUR cents 100 = CZK 24.565 (https://www.kurzy.cz/kurzy-men/historie/EUR-euro/2022/ (accessed on 24 September 2023)) was applied. ^2^ Costs expressed per average case of CM (treated both with and without antibiotics). ^3^ Ratio of CM diseases treated with antibiotics was constant over all lactations. ^4^ Period of discarded milk contained in the instructions for administering medication.

**Table 5:** Economic weights (EWs) and selection responses for the breeding objective traits applying selection on different enhanced SIH.

Parameter		Unit	Abbreviation	EWs (EUR/Unit)	Enhanced SIH Variants and Alternatives ^1^						
					(1) SIH + Udder		(2) SIH + Udder + Claw		(3) SIH + Udder +		
									Claw + Reproductive		
					opt	def	opt	def	opt	def	pref
Genetic Response		-	-	-	-	-	-	-	-	-	
Current	Milk yield per 305 d of lactation	kg	MY	0.268	79.17	51.25	78.33	49.63	78.97	48.65	51.6
	Milk fat content	%	%F	255.2	0.039	0.028	0.039	0.029	0.039	0.029	0.029
	Milk protein content	%	%P	520.1	0.001	0.013	0.002	0.014	0.002	0.014	0.013
	Conception rate of cows	%	CR	6.947	0.763	0.552	0.769	0.56	0.773	0.569	0.557
	Service period	days	SP	−0.023	−0.826	−0.682	−0.828	−0.683	−0.83	−0.688	−0.685
	Losses of calves in rearing	%	CL	−1.261	−0.031	−0.021	−0.03	−0.021	−0.03	−0.022	−0.021
	Age at first calving	days	AFC	−0.163	−1.712	−1.146	−1.707	−1.141	−1.716	−1.135	−1.150
	Mature weight of cows	kg	MW	−1.546	0.713	1.046	0.665	1.092	0.673	1.110	1.051
	Productive lifetime of cows	years	PL	139.3	0.169	0.072	0.169	0.073	0.168	0.074	0.072
	Clinical mastitis	case/cow year at risk	CM	−132.1	−0.004	−0.003	−0.004	−0.003	−0.004	−0.003	−0.003
New	Overall claw disease		OCD	−128.9			−0.004	−0.003	−0.004	−0.003	−0.002
	Retained placenta		RET	−52.10			-	-	−0.001	−0.001	−0.001
	Metritis		MET	−80.48			-	-	−0.006	−0.004	−0.004
	Cystic ovarian disease		CYS	−16.16			-	-	0.001	0.001	0.001
Total economic response ^2^		EUR	-	-	60	41	40	28	24	10	10
Reliability of the index		%	-	-	43.4	35.1	43.9	35.5	43.9	35.5	40

^1^ Index variants: (1) Present SIH for the Czech Holsten enhanced by clinical mastitis incidence (CD); (2) Index 1 enhanced by claw disease incidences (OCD, IDD, and CHL); (3) Index 2 enhanced by reproductive disorders (RET, MET, and CYS); Index alternatives for the index trait weighting: opt—optimised to maximise the selection response in the breeding objectives and the selection reliability; def—based on the proportion of traits equal to the current SIH index by default and making room for the new health disorder trait(s) from the optimal contribution; pref—the new traits taking into account breeders preferences. ^2^ Represents the sum of economic responses in all the breeding objective traits. The average annual exchange rate of EUR 1 = EUR cents 100 = CZK 24.565 was applied (https://www.kurzy.cz/kurzy-men/historie/EUR-euro/2022/ (accessed on 24 September 2023)).

## Data Availability

The data used in this study are the property of the farmers and Holstein Cattle Breeders Association of the Czech Republic and therefore cannot be publicly shared.
